# Controlling the dispersion of supported polyoxometalate heterogeneous catalysts: impact of hybridization and the role of hydrophilicity–hydrophobicity balance and supramolecularity

**DOI:** 10.3762/bjnano.5.185

**Published:** 2014-10-10

**Authors:** Gijo Raj, Colas Swalus, Eglantine Arendt, Pierre Eloy, Michel Devillers, Eric M Gaigneaux

**Affiliations:** 1 Institute of Condensed Matter and Nanosciences, Division MOlecules, Solids and reactiviTy, Université catholique de Louvain, Croix du Sud 2, L7.05.17, B-1348, Louvain-la-Neuve, Belgium; 2 CEA Grenoble, INAC, UMR 5819 SPRAM (CEA/CNRS/UJF-Grenoble 1), Laboratoire d’Electronique Moléculaire, Organique et Hybride, 17 rue des Martyrs, 38054 Grenoble cedex 9, France

**Keywords:** atomic force microscopy, heterogeneous hybrid catalyst, organic–inorganic hybrid materials, polyoxometalates, supramolecular organization

## Abstract

The hybridization of polyoxometalates (POMs) through an organic–inorganic association offers several processing advantages in the design of heterogeneous catalysts. A clear understanding of the organization of these hybrid materials on solid surfaces is necessary to optimise their properties. Herein, we report for the first time the organization of Keggin phosphotungstic [PW_12_O_40_]^3−^ and Wells–Dawson (WD) phosphomolybdic [P_2_Mo_18_O_62_]^6−^ anions deposited on mica (hydrophilic), and highly oriented pyrolytic graphite (HOPG) (hydrophobic) surfaces. Next, the supramolecular organization of the organic–inorganic hybrid materials formed from the association of POM anions and dimethyldioctadecylammonium bromide (DODA) is investigated as a function of the hydrophilic or hydrophobic nature of the surfaces. The height of the Keggin-POM anions, measured with tapping mode (TM-AFM) is always in good agreement with the molecular dimension of symmetric Keggin-POM anions (ca. 1 nm). However, the asymmetric WD-POM anions form monolayer assemblies on the surfaces with the orientation of their long molecular axis (ca. 1.6 nm) depending on the hydrophilic or hydrophobic properties of the substrate. Namely, the long axis is parallel on mica, and perpendicular on HOPG. When hybridized with DODA, the organization of the hybrid material is dictated by the interaction of the alkyl side chains of DODA with the substrate surface. On HOPG, the DODA–POM hybrid forms small domains of epitaxially arranged straight nanorod structures with their orientation parallel to each other. Conversely, randomly distributed nanospheres are formed when the hybrid material is deposited on freshly cleaved mica. Finally, a UV–ozone treatment of the hybrid material allows one to obtain highly dispersed isolated POM entities on both hydrophilic and hydrophobic surfaces. The hybridization strategy to prevent the clustering of POMs on various supports would enable to develop highly dispersed POM-based heterogeneous catalysts with enhanced functionalities.

## Introduction

Polyoxometalates (POM) are well-defined oxoanionic clusters of early transition metals that have attracted growing interest for the development of advanced functional materials. A plethora of tunable properties combined with their ability to form hybrid materials with organic moieties, have opened up new avenues not only in the field of supramolecular chemistry, but also in new materials technology [1–2]. For example, their tunable molecular architecture, charge density, strong redox capability, electro- and photochemical properties, make that these molecular metal-oxide nano-clusters are increasingly applied in diverse fields, such as medicine [3], magnetism [4–6], electronics [7], electro- and photochromic systems [8–9], and catalysis [10–14].

The hybridization of POM anions in an organic matrix offers several practical advantages. In catalysis, for example, hybridization offers a means to synthesize heterogeneous POM-based catalysts with enhanced processability, recoverability, and reusability. Organic–inorganic hybridization can be achieved either, through electrostatic interactions between POM anions and a polyampholyte polymer [15], or through covalent functionalization of POM anions with an organic moiety [16]. A fundamental understanding of the self-assembly and organization of these hybrid materials on solid surfaces is very important in order to optimise their use. Hence POM-based hybrids have been the subject of intense research in the past decade. The organization of the hybrid material on surfaces is dependent on the interaction between various factors such as the type of hybridization (i.e., through electrostatic interaction or covalent bonding), the nature of the POM building block (formula, charge density), and the chemical nature and hydrophilic or hydrophobic character of the surface on which the hybrids are deposited. For instance, surfactant-encapsulated clusters of POM anions, formed through electrostatic interactions, were reported to form well-ordered straight nanorods on graphite [17], whereas hybrid materials formed through covalent functionalization of POM formed planar layer-by-layer assemblies on a hydrophobic surface [16]. Recently Musumeci et al. [18] have shown the formation of nano-hexagons in a hybrid made from an alkyl-functionalised Mn-Anderson POM and dimethyldioctadecylammonium bromide (DODA) as counter cation. These hybrid nano-hexagons deposited on a SiO_2_ surface, through Langmuir–Blodgett technique, exhibited a high dielectric constant, which makes them promising candidates for applications in future memory storage, and hybrid (organic–inorganic) electronic devices.

Often cationic surfactants such as DODA were used as host organic matrix to synthesize organic–inorganic hybrid materials, with their organization having mostly been investigated on hydrophobic surfaces [17] or in bulk [19]. However, a clear understanding of their structure and molecular assembly as a function of the hydrophobic or hydrophilic nature of the substrate is absolutely necessary for further progress towards a wider application. In this study we, thus, systematically analyse these systems through tapping-mode atomic force microscopy (TM-AFM). First we investigated the organization of two kinds of POM anions, namely a Keggin phosphotungstic [PW_12_O_40_]^3−^ species, and a Wells–Dawson (WD) phosphomolybdic [P_2_Mo_18_O_62_]^6−^, deposited either on HOPG (hydrophobic) or on mica (hydrophilic) surfaces. Second, the supramolecular organization of the organic–inorganic hybrid materials made from POM anions and DODA were investigated as a function of the hydrophilic or hydrophobic nature of the surfaces on which they were deposited. Finally, we submitted the hybrid structures to an UV–ozone treatment to study the role of the alkyl chains in determining the well-defined supramolecular architectures of the organic–inorganic hybrids priorly observed.

## Experimental

### Reagents

DODA (C_38_H_80_NBr, *M* = 630.95 g·mol^−1^) was of analytical grade (Sigma-Aldrich) and used without further purification. The Keggin-type phosphotungstic POM was purchased from Sigma Aldrich in the acidic form (H_3_PW_12_O_40_, *M* = 2988.2 g·mol^−1^). Whereas, the Wells–Dawson POM was synthesized at our lab (please see “Preparation of Wells–Dawson POM”). Absolute ethanol (purity > 99.9%, VWR Belgium) was used as the solvent to prepare POM, DODA, and hybrid solutions.

#### Preparation of Wells–Dawson POM

The Dawson heteropolyanion [P_2_Mo_18_O_62_]^6−^ was prepared in its acidic form with the ethereal method. An amount of 5.00 g of (NH_4_)_6_P_2_Mo_18_O_62_ prepared as described in [20–22] was dissolved in 10 mL of distilled water. To this solution, 6 mL of concentrated HCl (37%, Sigma-Aldrich) was added. The acidified solution was transferred to a 1 L separating funnel, mixed with 20 mL of diethyl ether (Merck 99.7+%) and left standing. After 10 to 15 min, three layers were formed: an upper ether layer, an intermediate aqueous layer and a heavy oily layer. The separating funnel was cooled with tap water and the lowest layer was transferred to another separating funnel and shaken with 10 mL of distilled water. To the mixture, 6 mL of concentrated HCl and 20 mL of diethyl ether were added and the resulting solution was shaken again. After cooling, the lowest layer was again transferred to another separating funnel, and the above washing step with HCl and diethyl ether was repeated once more. Finally, the ethereal solution was transferred to a beaker containing 4 mL of distilled water. The resulting solution was evaporated on a water bath with occasional stirring until crystals began to form on the surface. The crystals were allowed to cool down to room temperature and were filtered through a Büchner funnel. Infrared and Raman spectra were used to identify the synthesized crystals by measuring the characteristic bands corresponding to the main Dawson units, and the purity of the prepared compound was confirmed by elemental and XPS analysis [23].

#### Preparation of thin films

A solution of DODA–Keggin POM hybrids was prepared by mixing DODA (1.58 × 10^−6^ mol·L^−1^) and phosphotungstic anion (0.53 × 10^−6^ mol·L^−1^) in absolute ethanol under magnetic stirring for 12 h. The molar ratio of 3:1 was kept for the DODA–Keggin POM hybrid solution in order to meet the charge balance between the POM anions (3 negative charges) and the DODA cation (1 positive charge). In the case of WD POM, the hybrid solution was prepared by mixing WD POM solution (0.26 × 10^−6^ mol·L^−1^) and DODA (1.58 × 10^−6^ mol·L^−1^) in ethanol under magnetic stirring for 12 h. In this case, the DODA/POM molar ratio was kept at 6:1 in order to meet the charge balance of WD, which bears 6 negative charges. Thin films of pure POM, pure DODA, and hybrid solutions were obtained by depositing a drop of each solution (about 9.5 µL) either on to freshly cleaved HOPG (ZYB grade, Bruker AFM probes) or mica surfaces (Agar scientific). The sample was allowed to dry under ambient conditions for more than 48 h before analysis. The DODA–POM hybrid films, after AFM imaging, were treated in an UV–ozone chamber (Jelight USA) for 10 min.

#### Atomic force microscopy

AFM experiments were performed by using a Nanoscope V multimode AFM (Bruker AXS) in tapping mode under ambient conditions (23 °C and 56% RH). Thin films of the POM, DODA, and the hybrid materials were imaged by using an etched Si tapping mode cantilever of the TESP type (Bruker AFM probes), having a nominal radius of curvature of 8 nm. Samples were glued on a magnetic stainless steel disc by using double-sided adhesive tape before mounting on to the “J” type piezoelectric scanner. The ratio of the set-point amplitude to the free amplitude was kept at 0.9 in order to apply minimal forces (“light tapping” conditions) so as to prevent sample deformation. Images were acquired at a resolution of 512 samples per line with a scan speed of 0.5 lines/s. Raw images were analysed by using the Nanoscope scan analysis software (Bruker) and flattened to the zeroth order to remove any underlying surface curvature.

#### X-ray photoelectron spectroscopy

X-ray photoelectron spectroscopy (XPS) analysis was performed on a Kratos Axis Ultra spectrometer (Kratos Analytical, Manchester UK) equipped with a monochromatised Al X-ray source (powered at 10 mA and 15 kV) and an eight-channeltrons detector. The samples were fixed on a standard stainless steel multi-specimen holder by using a piece of double-sided insulating tape. The pressure in the analysis chamber was approximately 10^−6^ Pa. The pass energy was set at 160 eV for the collection of the survey spectrum, and 40 eV for narrow scans of individual elements. In the latter conditions, the full width at half maximum of the Ag 3d 5/2 peak of a standard silver sample was about 0.9 eV. The Kratos Axis device was used for charge stabilisation.

## Results and Discussion

### AFM imaging of POM and DODA alone

**Figure 1:**
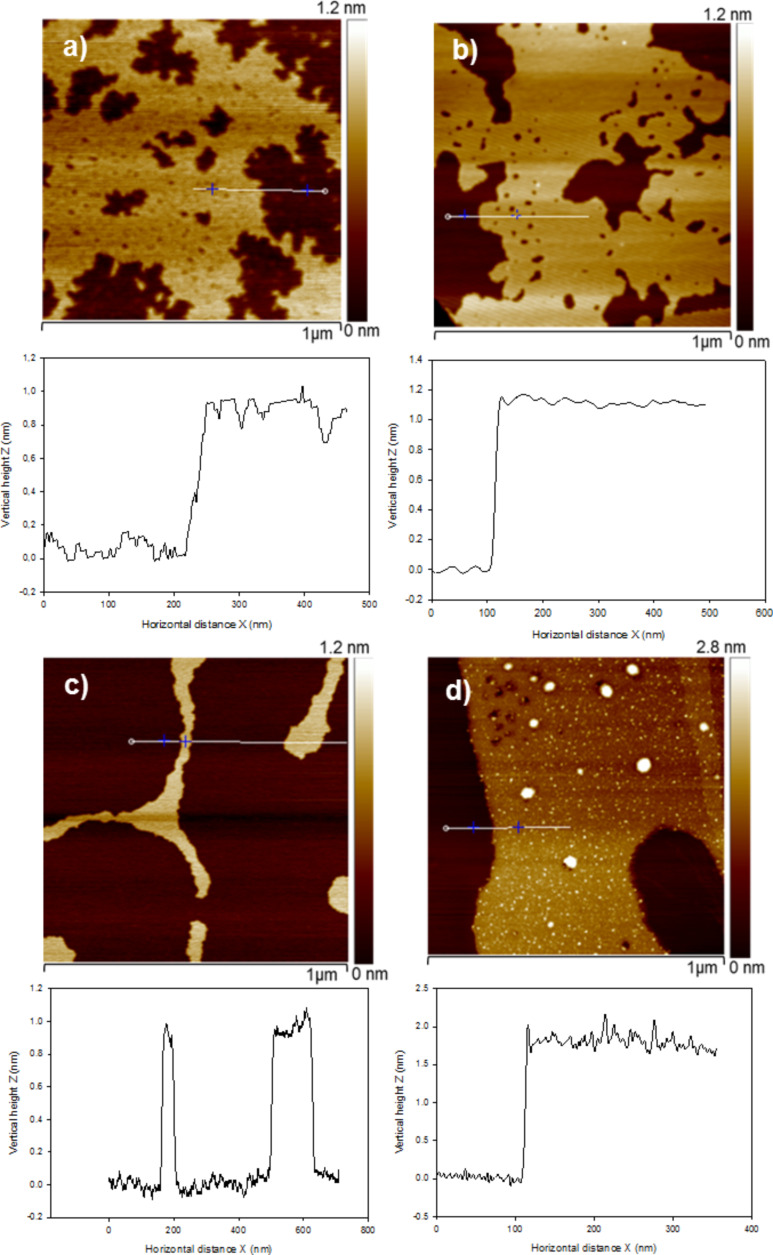
TM-AFM height images of Keggin POM (a,b), and Wells–Dawson POM (c,d) deposited on mica and HOPG surfaces, respectively. The corresponding cross-section analyses taken along the white lines are shown below each image.

The nano-structures of POM, and DODA, deposited alone on mica and HOPG surfaces were first investigated. Figure 1 shows the AFM images of the two different types of POMs used in this study. The Keggin POM form monolayers with a height of 1.0 ± 0.05 nm on both mica and HOPG surfaces, as measured by the cross-section analysis (Figure 1a and Figure 1b, respectively). This measured vertical height is in close agreement with the theoretical size (diameter of about 1 nm) estimated for the Keggin anion [PW_12_O_40_]^3−^ [24–25]. This suggests that the monolayers are indeed formed by self-assembled individual POM units.

**Figure 2:**
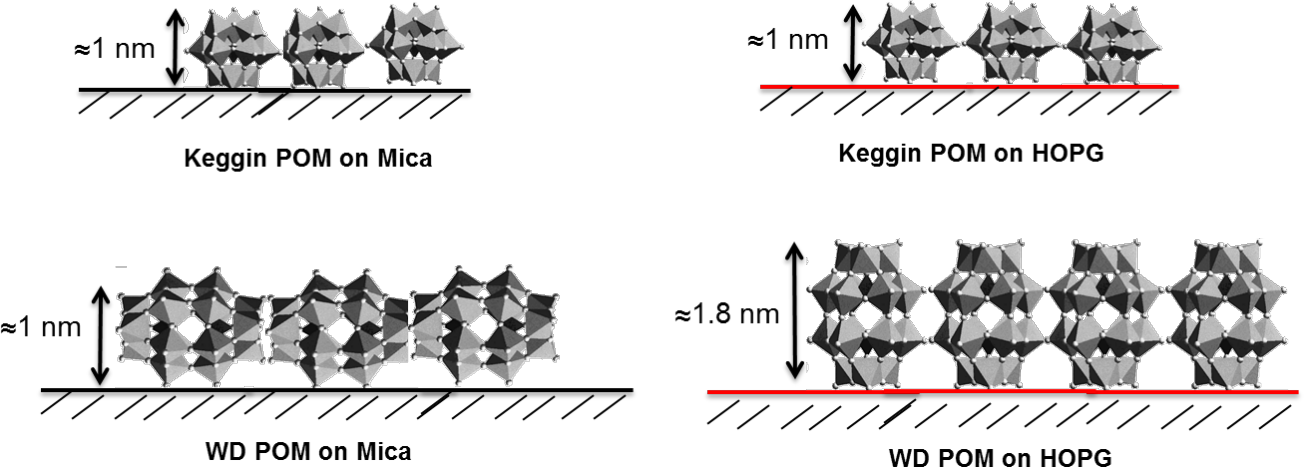
Schematic representation of the arrangement of Keggin and WD POM on mica and HOPG surfaces.

Wells–Dawson (WD) POM forms large island-like structures when drop casted on mica and HOPG surfaces. The height of WD-POM deposited on the mica surface was ca. 1 nm (Figure 1c), which is in close agreement with the short molecular axis of the asymmetric WD-POM (1.05 nm × 1.45 nm) [26–27]. On the other hand, the height of the WD-POM structures deposited on the HOPG surface was ca. 1.75 nm (Figure 1d), in close agreement with the long molecular axis of WD-POM. It could be noted that the concentration of POM appear to be low in some AFM images, e.g., Figure 1c, which is solely because of a local concentration gradient due to the slow evaporation of the solvent. For example, the effect of this local concentration gradient is shown in Figure S1 (Supporting Information File 1), in which regions of high and low concentrations of POM monolayers are clearly observed in a sample of WD POM on mica. The existence of a local concentration gradient, however, does not affect our conclusions since the results are based on the vertical height of the POM monolayers, which remains the same in both regions of low and high concentration. Thus, the difference in the height measurement is an indication of the preferential orientation of WD oxoanions on different surfaces. On the hydrophilic mica surface, they lay flat with the long molecular axis parallel to the surface, allowing for maximum contact of the hydrophilic sites of POM with the surface. Conversely, on hydrophobic HOPG, they form molecular assemblies with the long molecular axis perpendicular to the surface which results in minimum contact of the hydrophilic sites with HOPG. In the case of Keggin POM, however, it is interesting to note that the similar values of height measured on both mica and HOPG surfaces (ca. 1 nm) reflects the symmetrical dimension of the Keggin POM species. The preferential orientation of different kinds of POM as a function of the hydrophilic or hydrophobic nature of the support is illustrated in Figure 2. This finding has implications on practical applications, e.g., in catalysis in which the functionality (activity, selectivity and/stability) of POM can be tuned by controlling the preferential orientation on the support [28].

**Figure 3:**
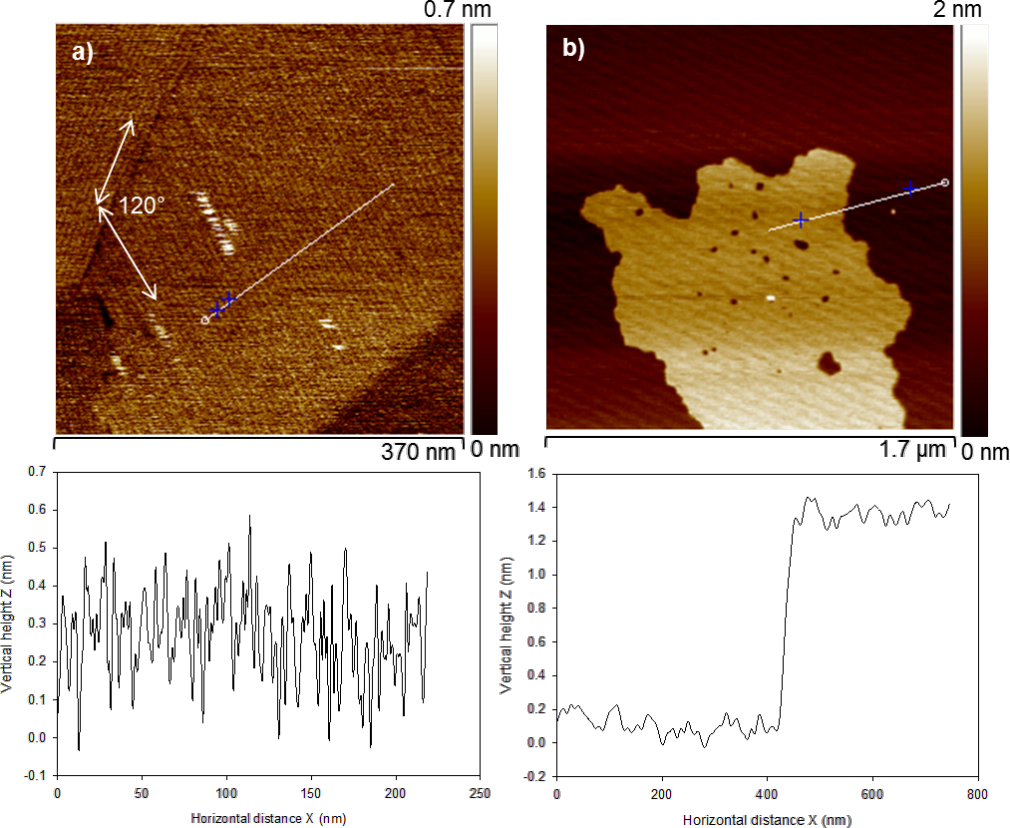
TM-AFM height images of DODA deposited on a) HOPG and b) mica. Corresponding cross-section analyses taken along the white lines are shown below each image.

Figure 3 represents the TM-AFM images of only DODA deposited on HOPG and mica. Domains of long, extended, and self-assembled chains of DODA (0.35 nm high), oriented at 120° were formed on HOPG (Figure 3a). The 120° angle coincides with the primitive hexagonal lattice structure of the underlying HOPG plane. Thus, the alkyl chains of the DODA are epitaxially arranged on HOPG surface. It is known that the close match of the C–C bond length (0.251 nm), and the crystallographic <1120> HOPG spacing (0.246 nm) favours an epitaxial growth of alkyl chains on HOPG, oriented along the <1120> lattice direction [29]. In contrast, DODA form small island structures of 1.5 nm height on mica (Figure 3b). On the hydrophilic mica surface, the alkyl chains of DODA are known to orient with a tilt angle of θ ≈ 30° with respect to the normal of the surface [30]. As expected, no epitaxial arrangement of DODA was observed. The conformation of the DODA surfactant, as shown here, can thus be tuned as a function of the surface properties, e.g. through hydrophobic or hydrophilic interactions or through epitaxial growth.

### Supramolecular organization of DODA–POM hybrids

**Figure 4:**
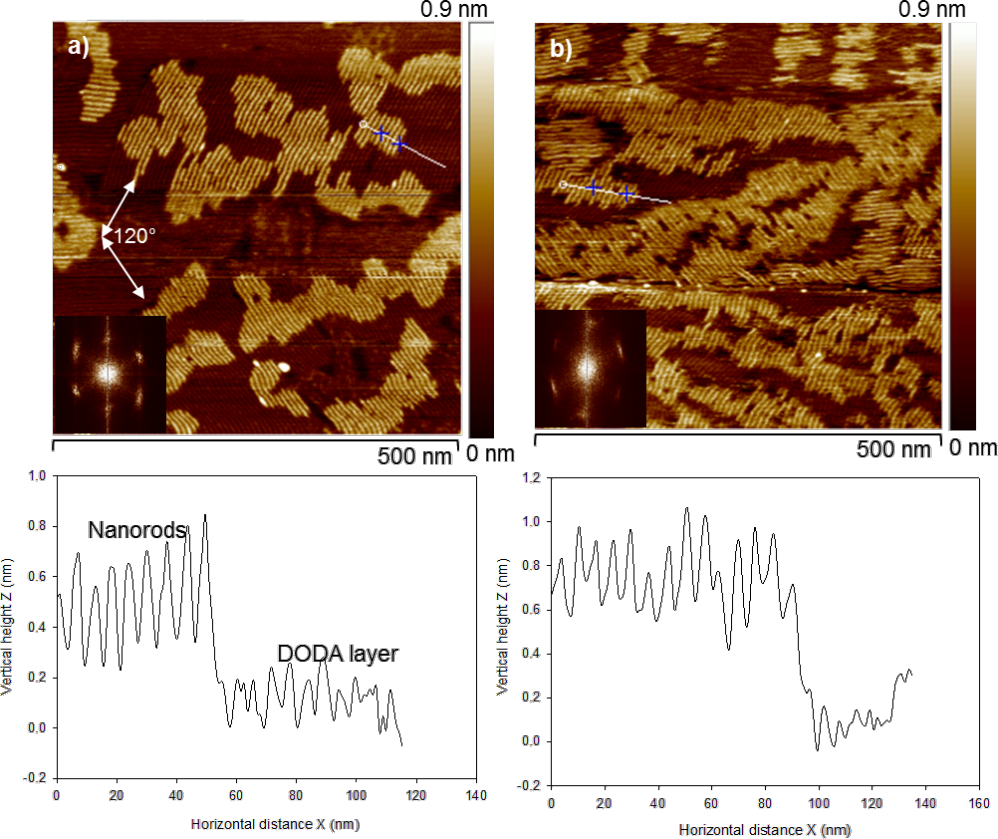
TM-AFM images of the different DODA–POM hybrids deposited on HOPG. a) Hybrids made from Keggin POM, b) Hybrid made from WD-POM. Insets show 2DFFT images. Cross-section analyses taken along the white lines are shown below each image.

In the following section, we discuss the supramolecular organization of DODA–POM hybrids deposited on different surfaces. Hybridization of POM and DODA is expected to be driven through the electrostatic interaction of POM anions with the positively charged DODA surfactant. Firstly, we study the DODA–POM hybrids deposited on HOPG. Drop-casting of a small amount (about 9.5 µL) of a very dilute solution (0.025 g·L^−1^) allows for the formation of molecular layers that are of particular interest to observe any self-assembled features. DODA–Keggin POM, and DODA–WD POM hybrids form small domains scattered over the HOPG surface. High resolution AFM images of these small domains reveal that they are composed of well-ordered straight nanorod like features oriented parallel to each other (Figure 4). The domains of self-assembled nanorods were oriented at about 120° toward each other. The 2-dimensional fast Fourier transformation (2D-FFT) analysis of the images revealed three pairs of bright spots arranged in a three-fold rotational symmetry. These resemble the hexagonal arrangement of carbon atoms in the graphite basal plane, and hence reveal that the orientation of nanorods in the hybrid material is indeed controlled by the epitaxial interaction of DODA chains with HOPG.

The periodicity of the nanorod arrays, measured as peak-to-peak distances from AFM cross-section analysis, was 6.38 ± 0.05 nm, and 6.5 ± 0.05 nm for the DODA–Keggin POM and DODA–WD POM hybrids, respectively (Figure 4). The theoretical size of a single Keggin anion [PW_12_O_40_]^3−^ is ca. 1 nm [31], and the length of a fully extended DODA molecule is ca. 2.25 nm [17]. Because the POM structures used in this study do not possess a ring structure that can hold water molecules [19], and because we have used a dehydrated solvent (ethanol), the theoretical size of a DODA encapsulated single Keggin anion unit can be estimated to be roughly 5.5 nm. The estimation of the (lateral) diameter of the nanorods by AFM is semi-quantitative due to the well-known tip-convolution effect [32]. However, the (vertical) peak-to-peak distance of periodic structures measured by AFM is more accurate and it closely matches with the theoretical size of the hybrid nanorods.

The formation of the nanorods was also determined by the molar ratio of DODA:POM in the hybrid. For example, in the case of the Keggin POM, perfect nanorod assemblies were obtained when DODA and POM were mixed in the ratio of 3:1, i.e., when the charges on both species were compensated in the hybrid material. However at high POM loadings, e.g., a DODA/POM ratio of 1:3, instead of the nanorods, randomly distributed large clusters of POM entities were formed on HOPG (Figure S2a, Supporting Information File 1). On the other hand, at high DODA loadings (DODA/POM ratio 6:1), hybrid nanorods were formed over a template layer of DODA on HOPG (Figure S2b, Supporting Information File 1).

**Figure 5:**
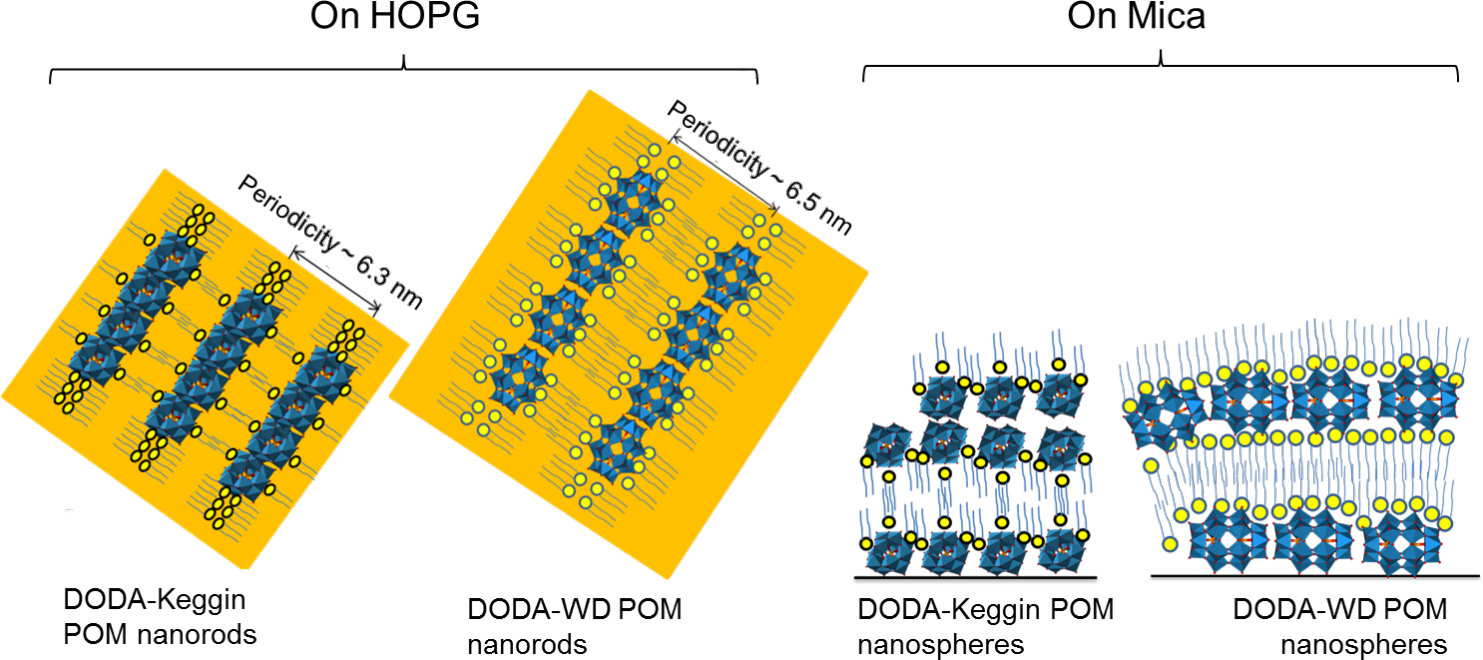
Supramolecular organization of DODA–POM hybrids on HOPG and mica surfaces.

Molecular modelling by Polarz et al. [19] show that the DODA–POM hybrids consist of defined units, in which the cationic surfactant has all protons replaced and is strongly attached to the POM cluster. However, a close examination of our AFM images shows that the nanorods are indeed formed on an already well-organised underlying layer (Figure 4a). The cross-section analysis of the underlying layer together with the nanorods, shows differences in the peak-to-peak distances. The peak-to-peak distance of the underlying layer was close to that of the periodicity of the lamella arrays of DODA alone on HOPG, (ca. 4.8 nm). Thus it can be claimed that the DODA chains already form an epitaxially oriented template layer, onto which the organic–inorganic hybrid assemblies were grown. The template layer of DODA can also be seen in the case of nanorods made from WD POM (Figure 4b). However, the high number density of the nanorods here is mostly attributed to the local concentration gradient resulting from the slow evaporation of the solvent. A schematic of the nanorods assembly is shown in Figure 5. At the 3:1 molar ratio of DODA and POM in the solution, it is expected that the cationic surfactant substitutes all the protons in POM. However, when deposited on HOPG, the evidence of an underlying DODA layer indicates that either i) a complete substitution of protons with DODA might not had taken place, or ii) some of the DODA units could be detached from the hybrid structure due to the strong epitaxial interaction of alkyl chains of DODA with HOPG.

The vertical height of the nanorods, measured by AFM cross-section analysis, was 0.8 ±0.05 nm, and 1.1 ± 0.06 nm for the hybrids made from Keggin and WD POM, respectively. The heights of the hybrid assemblies were smaller when compared to the theoretical height of POM and DODA combined. A similar reduction in the vertical height of surfactant encapsulated clusters (SECs) of POM on HOPG, was also observed by Wu et al. [17] for their hybrid made of DODA and a larger (1.7 nm diameter) doughnut-shaped Preyssler anion [Na(H_2_O)P_5_W_30_O_110_]^14−^. The reduction in the vertical height of the nanorods was attributed to a flattening effect due to the interactions between flexible alkyl chains and the graphite surface. The small height value of the nanorods (equivalent to the height of a single POM), when compared to its large width (equivalent to the size of one POM and two DODA combined), is in agreement with our previous assumption that some DODA units might have detached from the hybrid structure due to the strong epitaxial interaction of alkyl chains of DODA with the HOPG.

**Figure 6:**
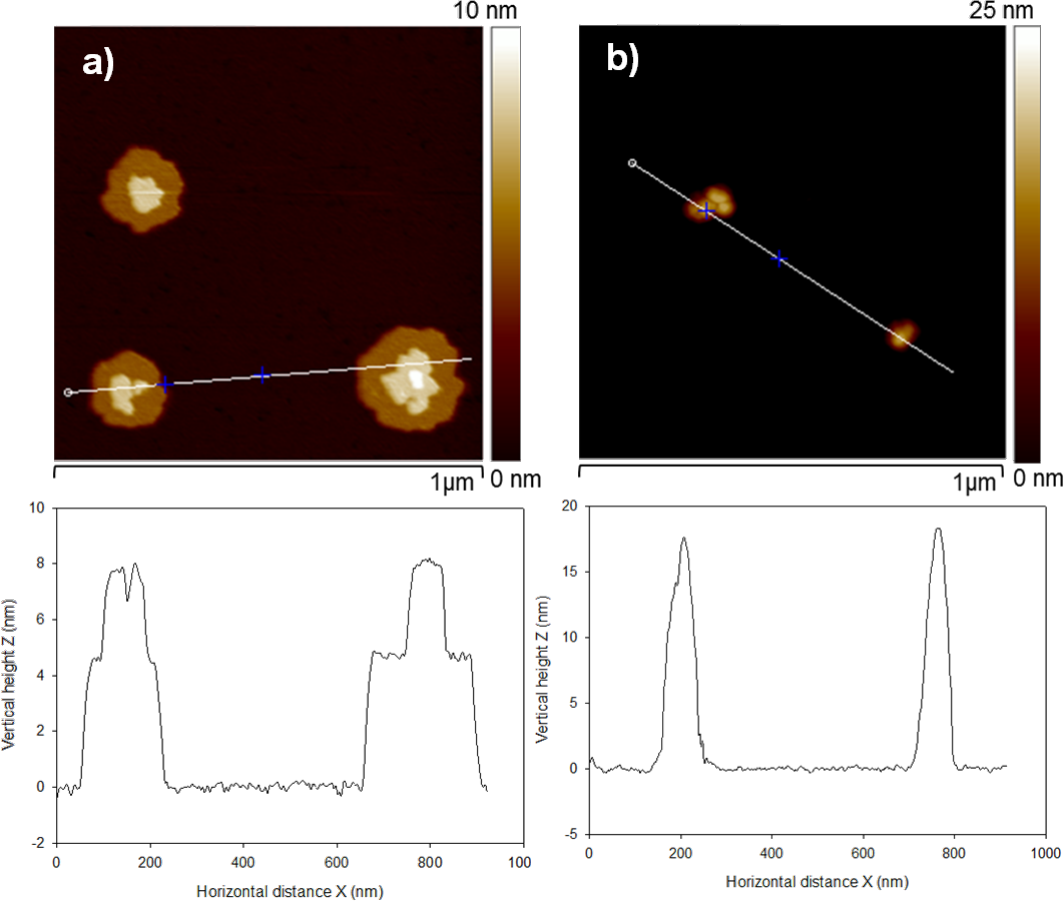
TM-AFM images of the different DODA-POM hybrids deposited on mica surface. a) Hybrids made from Keggin POM (height 8.2 nm), and b) Hybrid made from WD-POM (height 17.5 nm). Cross-section analysis taken along the white line is shown below each image.

In contrast to the self-assembled nanorods formed on HOPG, DODA–POM hybrids form randomly distributed nanosphere-like assemblies on the hydrophilic mica surface. The vertical height of the nanospheres, as measured by AFM cross-section analysis, were 8.0 ± 0.2 nm, and 17.5 ± 0.5 nm for the hybrids formed from Keggin and WD POM, respectively (Figure 6). A close examination of the cross-section of the nanospheres in Figure 6a shows that they are composed of vertical multi-layered assemblies. The thickness of the first layer is ca. 4.5 nm, while that of the second layer is ca. 3.5 nm, resulting in an overall thickness of ca. 8 nm. The vertical assembly of DODA–POM with a nanosphere structure is shown in Figure 5. It is interesting to note that the overall size of the nanospheres could be related to the size, as well the number of charges present on the POM anions. As the number of negative charges on the POM anions increased from 3 to 6, for the Keggin and WD POM, respectively, the total height of the hybrid assembly nearly doubles. This is also an indication that the organic–inorganic hybridization is largely dependent on the strength of electrostatic forces.

**Figure 7:**
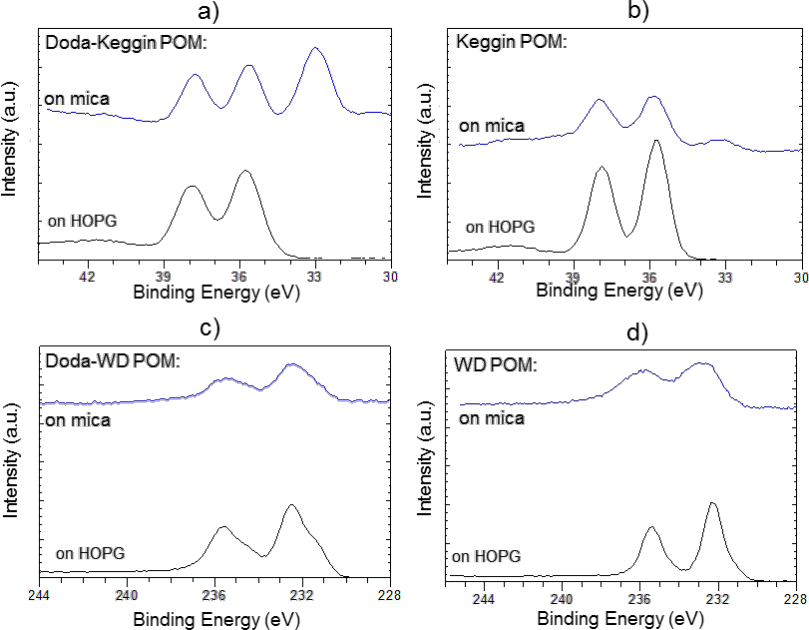
XPS analysis showing the core levels of different POMs and DODA–POM hybrids deposited on mica and HOPG surfaces. a) and b) are W 4f (37 and 35 eV) core levels of DODA–Keggin POM hybrid, and pure Keggin POM, respectively. c) and d) are Mo 3d (232 and 235 eV) core levels of DODA–WD POM hybrid and pure WD POM, respectively.

In order to further understand the hybrid materials, we were motivated to identify the POM anions in the encapsulated nanostructures through chemical as well as physical methods. In a chemical analysis, XPS was performed on the nanorods and nanospheres to analyse the presence of elements in these hybrid structures. Doublets corresponding to W 4f (37 and 35 eV) and Mo 3d (232 and 235 eV) core levels were identified for the DODA hybrids formed from Keggin and WD POMs, respectively (Figure 7). In the case of DODA–Keggin POM hybrids deposited on mica, the W 4f doublet is partially overlapped with an additional peak at 33 eV, corresponding to the 3s core level of potassium (K), present on the mica surface (Figure 7a). The presence of the K peak is an indication that much of the mica surface is exposed and hence the nanospheres formed are more isolated entities. However, the intensity of the K peak is reduced when pure Keggin POM species are deposited on mica, indicating a good coverage on the surface. The XPS results corresponds well to the AFM images that show a monolayer coverage of Keggin POM, and isolated nanosphere entities formed on the mica surface. Because we do not observe any isolated POM species in the AFM images of the hybrid nano-structures, and because the XPS signals of the elements were obtained by the collection of electrons coming from a few nm (about 10 nm) down the surface, we can unambiguously state that the POM anions were present in the nanorod and nanosphere structures formed on HOPG and mica, respectively.

**Figure 8:**
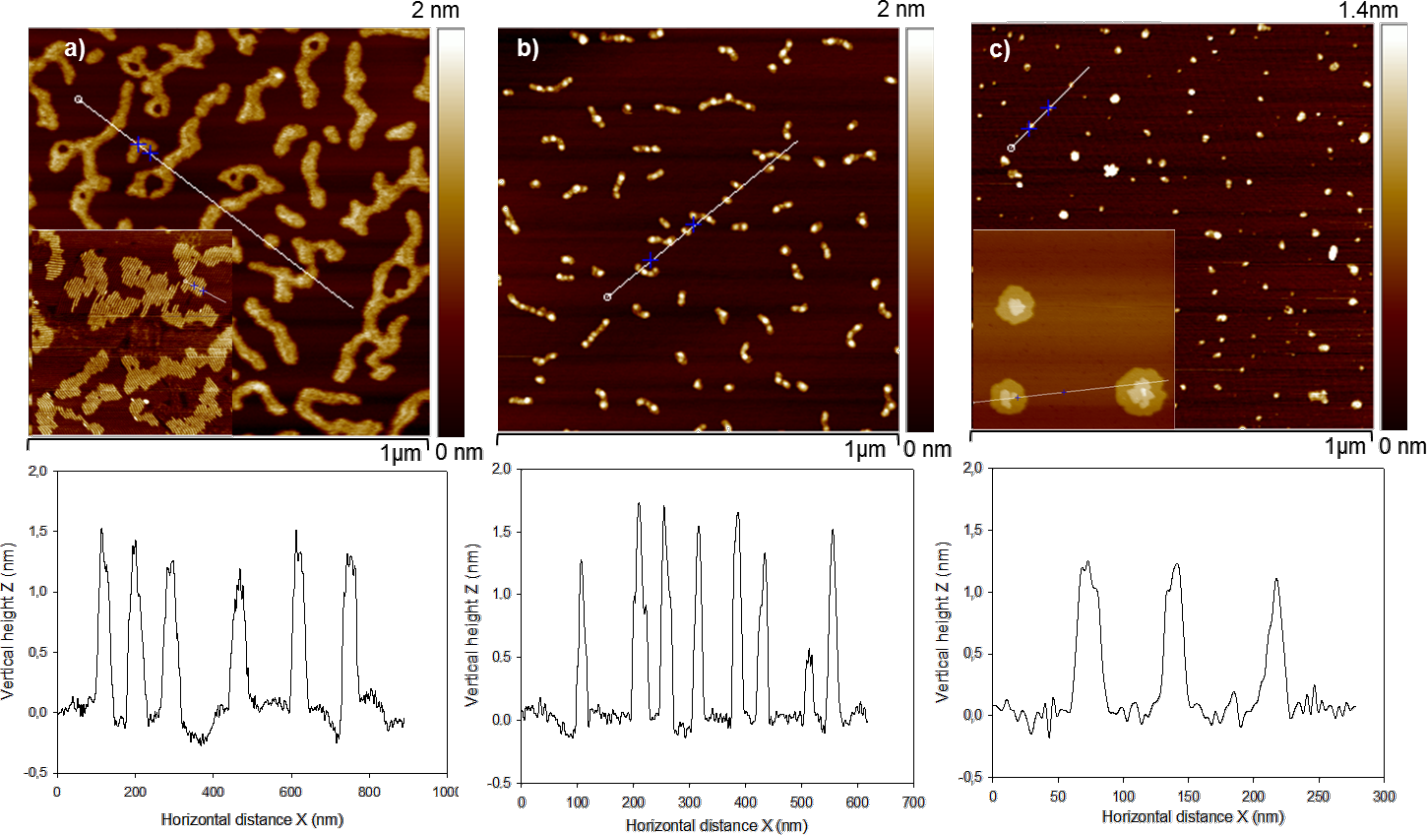
TM-AFM images of the UV–ozone-treated DODA–Keggin POM hybrid deposited on HOPG. a) Treatment time 1 min (inset shows the initial nanorod structure of the hybrid), b) treatment time 10 min, and c) UV–ozone-treated (10 min) DODA–Keggin POM hybrid deposited on mica (inset shows the initial nanosphere structure of the hybrid film on mica). Cross-section analyses taken along the white lines are shown below each image.

Finally, we submitted the hybrid nanostructures to an UV–ozone treatment in order to eliminate the hydrophobic chains and access the encapsulated POM anions. In a typical example, the DODA–Keggin POM nanorods formed on HOPG were treated under UV–ozone for different durations. TM-AFM images show that even one minute of UV–ozone treatment was sufficient to alter the epitaxial arrangement of the nanorods, leading to randomly oriented elongated structures, made mostly of self-assembled POM species (Figure 8a). After ten minutes of treatment, the elongated structures disintegrated into smaller units having a vertical height comparable to that of individual Keggin-POM units (Figure 8b).

Contact angle measurements were performed in order to verify the effect of UV–ozone treatment on the hydrophobic property of HOPG. Table S1 (Supporting Information File 1) shows that water contact angle decreased from 62.5 to 25° when the UV–ozone treatment duration was varied from 1 to 10 min, respectively. Thus, it is important to verify whether the high dispersion of POM entities on HOPG, after the UV–ozone treatment of the hybrid, is linked to the more hydrophilic nature that HOPG has acquired following this treatment. Hence DODA–POM hybrids were deposited on hydrophilic HOPG surface that was previously treated for 10 min under UV–ozone. AFM images indicate that the hybrid material forms island-like structures of 0.85 nm height (Figure S3, Supporting Information File 1). It is interesting to note here that, unlike on the fresh hydrophobic HOPG, the hybrid material does not form nanorods when deposited on the UV–ozone-treated HOPG. Another experiment was the UV–ozone treatment (10 min) of the above island-like structures. After the treatment, large POM clusters were observed similar to the case in which POM were deposited on fresh HOPG. This is in contradiction to the formation of highly dispersed POM entities formed after the UV–ozone treatment of the nanorods. Thus, it is the epitaxial interaction of the alkyl chains of the hybrid material with the HOPG, and not the hydrophilic nature that HOPG has acquired after the UV–ozone treatment, that plays a major role in the high dispersion of POMs on HOPG. On the mica surface, following the UV treatment for 10 min, the hybrid nanosphere structure collapsed and individual POM species appear to be dispersed on the surface (Figure 8c). This suggests that the UV–ozone treatment of the DODA–POM hybrid material could be an effective strategy to obtain highly dispersed isolated POM entities on both hydrophilic and hydrophobic surfaces, in contrast to the large island clusters formed when only POM ions were deposited on these surfaces. These findings have implications on practical applications, e.g., on the design heterogeneous catalyst design, in which the classical wet impregnation technique often leads to the formation of large crystallites, particularly at high POM loadings, on various inorganic and/or on hydrophobic supports [28]. The demonstrated hybridization strategy of POMs with DODA and the subsequent elimination of the surfactant molecules could lead to the development of highly dispersed POM heterogeneous catalysts with enhanced functionalities.

## Conclusion

We have demonstrated the role of hydrophobic and hydrophilic surface forces in determining the supramolecular organization of polyoxometalates (Keggin [PW_12_O_40_]^3−^, and Wells–Dawson [P_2_Mo_18_O_62_]^6−^), as well as their organic–inorganic hybrid materials formed from dimethyldioctadecylammonium bromide (DODA) on solid surfaces. The height of the POM anions measured by AFM was in good agreement with their molecular dimensions. The orientation of the long molecular axis of an asymmetric WD POM anion was found to be either parallel, or perpendicular depending on the hydrophilic or hydrophobic nature, respectively, of the substrate. This finding has implications on practical applications, e.g., on catalysis, where it offers a means to tune the functionality of WD POM according to its preferential orientation on surfaces. However, when POM was hybridized with DODA, the organization of the hybrid material was dictated by the interaction of the alkyl side chains of DODA with the substrate. The hybrid material form well-defined, straight and epitaxially arranged nanorod structures on a HOPG surface, while randomly distributed spheres were formed on mica surface. The pivotal role of the alkyl chains in maintaining the nanostructures was evidenced by the fact that these structures collapsed when the alkyl chains were eliminated by an UV–ozone treatment, which resulted in the formation of highly dispersed POM entities on both hydrophilic and hydrophobic surfaces. Our study not only highlights the role of hydrophilic or hydrophobic surface forces in tuning the supramolecular architecture of DODA–POM organic–inorganic hybrid materials, but also demonstrates that this hybridization strategy can be an effective means to obtain highly dispersed isolated POM units, which have important practical applications. This hybridization strategy could be used, for example in heterogeneous catalysts design, to obtain highly dispersed POM active species on various hydrophilic and hydrophobic supports.

## Supporting Information

File 1 Additional experimental data.
