# Supplementary material for: Controlling the dispersion of supported polyoxometalate heterogeneous catalysts: impact of hybridization and the role of hydrophilicity–hydrophobicity balance and supramolecularity
Source: Beilstein J Nanotechnol. 2014 Oct 10;5:1749–59. doi: 10.3762/bjnano.5.185 (PMC4222433; doi:10.3762/bjnano.5.185)
Supplement: File 1 — Additional experimental data. [file Beilstein_J_Nanotechnol-05-1749-s001.pdf]

Supporting Information

for

Controlling the dispersion of supported polyoxometalate heterogeneous catalysts: impact of hybridization and the role of hydrophilicity–hydrophobicity balance and supramolecularity

Gijo Raj*[1,2], Colas Swalus[1], Eglantine Arendt[1], Pierre Eloy[1], Michel Devillers[1] and Eric M. Gaigneaux*[1,§]

Address: [1]Institute of Condensed Matter and Nanosciences, Division MOlecules, Solids and reactiviTy, Université catholique de Louvain, Croix du Sud 2, L7.05.17, B-1348, Louvain-la-Neuve, Belgium and [2]CEA Grenoble, INAC, UMR 5819 SPRAM (CEA/CNRS/UJF-Grenoble 1), Laboratoire d'Electronique Moléculaire, Organique et Hybride, 17 rue des Martyrs, 38054 Grenoble cedex 9, France

Email: Gijo Raj - raj.gijo@gmail.com; Eric M. Gaigneaux - eric.gaigneaux@uclouvain.be

[§]TEL: +32-10-473665; Fax: +32-10-473649

Additional experimental data

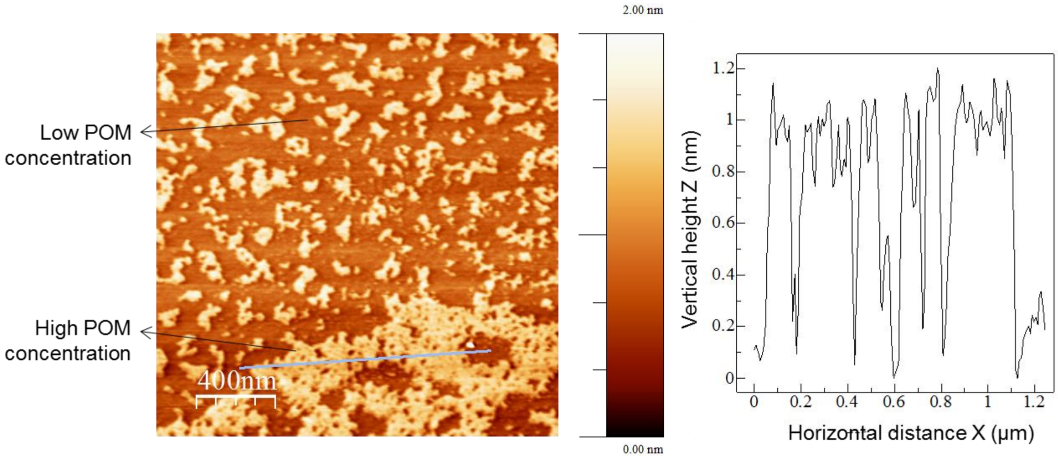

Figure S1: AFM height images of WD POM deposited on a freshly cleaved mica surface. Local concentration gradient results in regions with low and high concentrations of POM monolayers. The corresponding cross-section analysis shows that the POM monolayers have a height of ca. 1 nm.

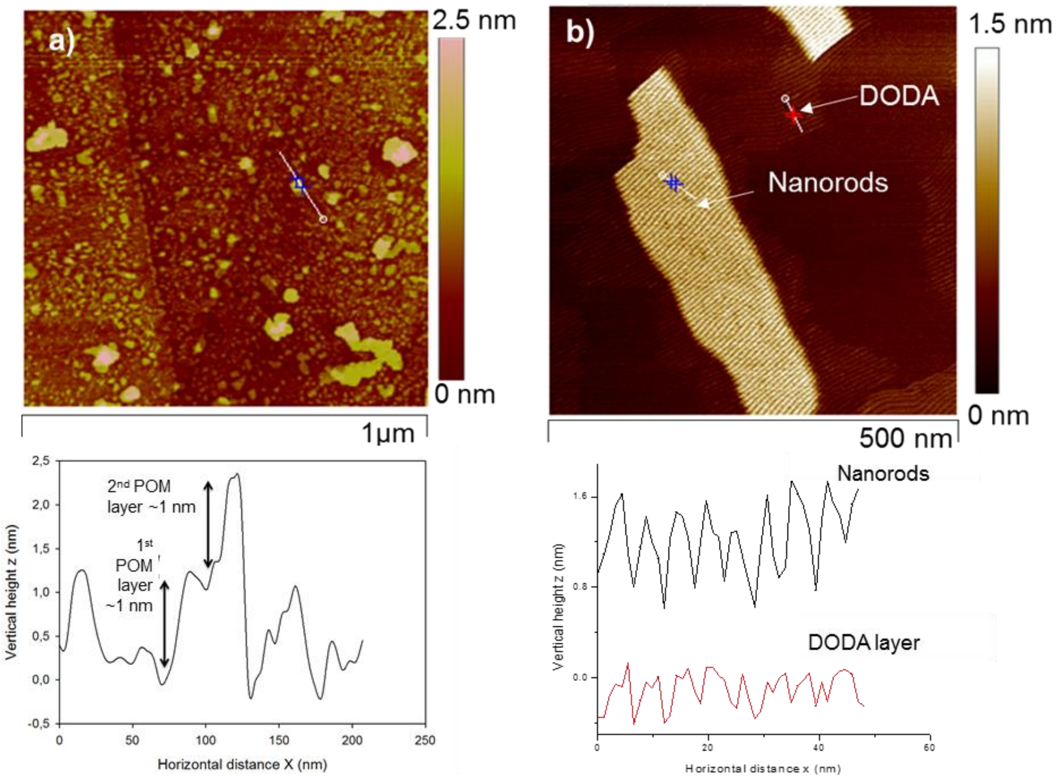

Figure S2: a) AFM height images of DODA–Keggin POM hybrids deposited on HOPG. a) Hybrid nanorods are not formed at high POM loadings (DODA/POM molar ratio of 1:3), but POM form clusters composed of 1 nm high units. b) Hybrid nanorods are formed on a self-assembled template layer of DODA at high DODA loadings (DODA/POM molar ratio of 6:1). Corresponding cross-sections taken along the white lines are shown below each image.

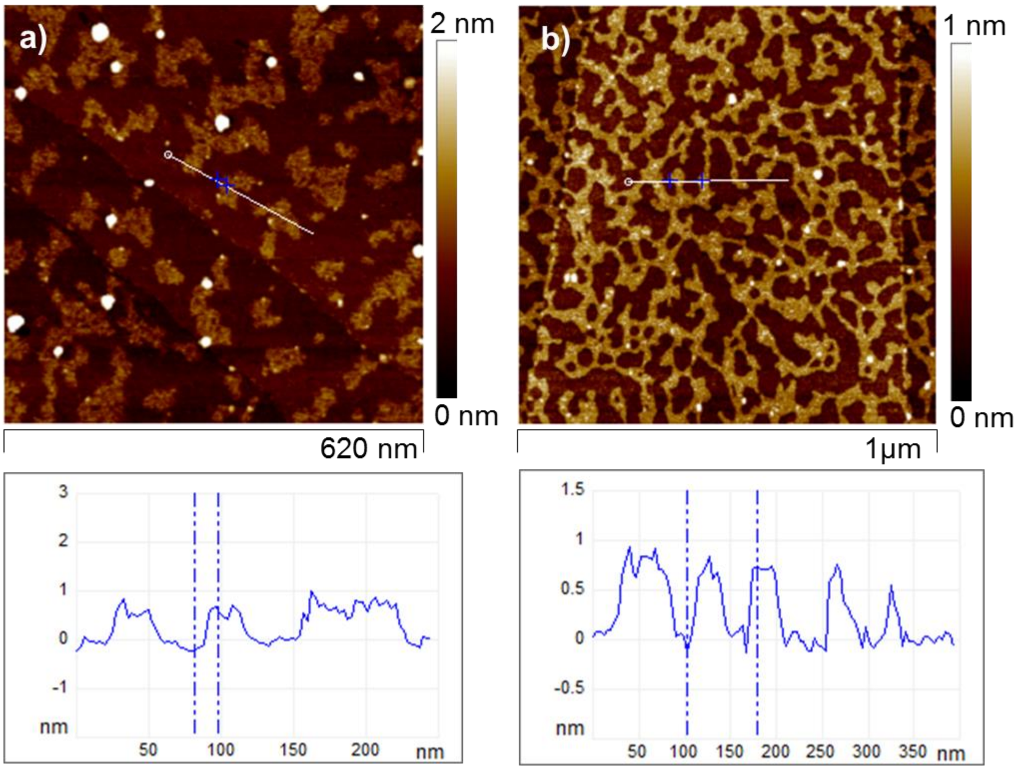

Figure S3: a) AFM height images of DODA–POM hybrids (DODA/POM molar ratio of 3:1) deposited on UV–ozone-treated HOPG (10 min). b) AFM height image show large clusters of POMs after UV–ozone treatment of a) for 10 min. Corresponding cross-sections taken along the white lines are shown below each image.

Table S1: Water contact angle measurements of HOPG surface after UV–ozone treatment.

substrate	water contact angle (θ)°
freshly cleaved HOPG	62.5 ± 1
HOPG after UV–ozone treatment (1 min)	61± 1
HOPG after UV–ozone treatment (10 min)	25± 1
